# Social inequalities in rural oral health: social capital and SDOH according to the severity of dental caries in Peru

**DOI:** 10.3389/fpubh.2026.1824635

**Published:** 2026-05-29

**Authors:** Juana Rosmeri Salas-Huamani, Roberto Antonio León-Manco, César Del Castillo-López, Taís de Souza Barbosa

**Affiliations:** 1 Facultad de Ciencias de la Salud, Escuela de Estomatología, Universidad César Vallejo, Lima, Peru; 2 Facultad de Estomatologia, Universidad Peruana Cayetano Heredia, Lima, Peru; 3 Departamento de Odontologia Social e Clínica Infantil, Instituto de Ciência e Tecnologia, Campus São José dos Campos, Universidade Estadual Paulista (UNESP), São José dos Campos, SP, Brazil

**Keywords:** children, dental caries, rural health, social capital, social determinants of health, oral health, social interaction

## Abstract

**Objective:**

To evaluate the association between Social Determinants of Health (SDOH), Social Capital (SC), and dental caries experience according to severity in 12-year-old children from a rural Peruvian community.

**Methods:**

A cross-sectional study was conducted with 61 schoolchildren and their families in Pampacolca, Arequipa - Peru. DMFT index (decayed, missing, and filled teeth) was assessed via clinical examination (kappa = 0.90). SDOH and SC were evaluated through household interviews with parents, using a survey based on the National Census and the short version of the Adapted Social Capital Assessment Tool (SASCAT), respectively. Data were analyzed using Spearman’s correlation and the coefficient of determination (R^2^).

**Results:**

The mean DMFT score was 5.25 ± 2.94. SDOH explained 3.99% of the variance in caries experience overall (*p* > 0.05). Significant associations were found when stratified by severity: in children with low severity (DMFT≤3), maternal education showed a significant correlation (*r* = −0.632, *p* = 0.007), explaining 39.9% of the variance. In children with high severity (DMFT≥5), limited access to health services was the most relevant factor (*r* = −0.605, *p* = 0.001), explaining 36.6% of the variance. Regarding social capital, only Cognitive SC showed a significant correlation (*r* = 0.494, *p* = 0.044), explaining 24.4% of the variance in the low-severity group.

**Conclusion:**

The findings suggest that SDOH and specific dimensions of SC are associated with dental caries, with effects varying by disease severity. While limited access to health services was linked to greater caries experience in high-severity cases, higher maternal education and cognitive social capital appeared to be associated with lower caries experience in children with lower severity. These results highlight the importance of context-specific social interventions.

## 1 Introduction

Dental caries continues to be a public health problem with a high prevalence in the child population, affecting their well-being, school performance and quality of life. In Latin America, social inequalities aggravate their impact, especially in rural areas with limited access to health services (1, 2). Social determinants of health (SDOH), such as economic, educational, and environmental conditions, directly influence the risk of developing oral diseases. In rural communities, limitations in access to fluoridated water, dental care, and health education increase children’s vulnerability (2–4).

Several studies have suggested that the social and community environment can modulate the distribution and severity of dental caries in children (3–5). However, current evidence is still insufficient to explain why children living under the same precarious social determinants exhibit different levels of disease severity, suggesting that unmeasured community-level factors, such as social capital (SC), may modulate these outcomes.

Social capital, understood as the set of networks, norms, and social trust that facilitate cooperation and collective well-being, has shown positive associations with various health indicators. Nevertheless, its relationship with childhood oral health remains underexplored, especially regarding its potential to compensate for the lack of formal health infrastructure (5). Conceptually, social capital may influence oral health by facilitating the rapid diffusion of health information, strengthening informal support networks for dental hygiene, and increasing collective efficacy to manage common resources (3–5).

The academic literature has yet to determine whether community assets can effectively buffer the impact of structural poverty or if they are overshadowed by it. In the context of Arequipa, Peru, this relationship has not been documented, representing a significant gap in regional public health knowledge. Understanding how social capital and SDOH influence caries severity will allow for the design of more comprehensive and community-based interventions. Therefore, the present study aims to evaluate the association between social capital and SDOH according to the severity of caries in children from a rural area in Arequipa, Peru.

## 2 Materials and methods

Observational, descriptive, cross-sectional and prospective study.

### 2.1 Ethical aspects

The research project was registered with SIDISI number 59474 and was presented for evaluation to the Methodological Committee and the Institutional Ethics Committee of the Universidad Peruana Cayetano Heredia, which approved the execution of the project, informed consent, informed assent and realization of the pilot test.

### 2.2 Calibration and pilot test

The examiner was calibrated in the evaluation of dental caries experience with the DMFT Index through theoretical training, calibration in mock-ups and calibration in patients in charge of a Gold Standard, obtaining a kappa value of 0.9 in the inter-examiner and intra-examiner calibration in the two calibrations. A pilot test was carried out that made it possible to standardize the techniques, test the adequate collection of information with the surveys for the established variables and improve the procedures for the execution of the project itself.

The pilot test was carried out in a community with characteristics like Pampacolca (Piscopampa annex, located 10 km from Pampacolca, Arequipa - Peru) with the participation of 15 12-year-old children and their respective mothers. Being a rural community, some important aspects were evaluated, it was observed that the routine of the families and the times when they were at home available to be able to respond to the surveys that were intended to be carried out was at the end of the afternoon from 5 p.m. since during the day they were dedicated to feeding their animals, work in the fields and activities outside the home. In addition, it was observed that the most present person during these hours was the mother of the family since the father was usually away from home, slept in the field taking care of the animals or had a job several hours from his city and only returned home on the weekend. According to these schedules, it was possible to visit 2 to 3 houses per day since the clinical examination of the 12-year-old boy or girl would be at the school in the morning period, where his address was recorded and after consent and signed assent, visits were made to their homes to fill out the surveys of both social determinants of health and social capital. This visit would take approximately 40 to 60 min per family. The pilot test basically served to estimate the appropriate times, adapt to the schedules of the families and prepare all the materials for the execution of the project.

### 2.3 Population and sample

The population was made up of 12-year-old children from the District of Pampacolca, Province of Castilla, Department of Arequipa. It was estimated that the real volume of subjects of this age would be 82 individuals according to the 2007 National Census: XI of population and VI of Housing (INEI), the data collected from the census was 8 years of age that according to projections would be 12 years old by the year of the study. A convenience sample was used, aiming to reach all families with children aged 12 and over. Given the lack of medical and dental services in this rural community, the two-hour distance to the nearest health center, and the ten-hour distance to the nearest dentist by land, these characteristics, along with the residents’ varied schedules, define this rural community as one that is difficult to access. The sample consisted of 61 12-year-old children from the Juan Pablo Vizcardo y Guzmán Public School. The selection criteria were: 12-year-old children from the District of Pampacolca who do not have systemic aggravating factors: diabetes mellitus, tuberculosis, malnutrition, acquired immunodeficiency syndrome, epilepsy, children who do not have congenital anomalies of the oral cavity: bilateral cleft lip, unilateral, etc. Children who do not have any type of mental syndrome or disability, children who do not collaborate with the collection of information or parents or guardians of individuals who do not collaborate with the collection of information.

### 2.4 Variables and instruments

- Social determinants of health: The survey based on the 2007 National Population Census and the VI Housing Census was used to measure the social determinants of health in families.
- Social capital: Short version or the Adapted Social Capital Assessment Tool (SASCAT) [Validated in Peru by De Silva et al. (6) and validated by experts by Manco (7), to measure the social capital of families].
- Experience of dental caries: Epidemiological file generated by the Academic Department of Social Dentistry of the Universidad Peruana Cayetano Heredia to measure the severity of the prevalence of dental caries in 12-year-old children.

### 2.5 Data collection techniques and procedures

The children’s oral exams were performed during school hours, in classrooms with natural light. The participant was placed in a chair facing the source of natural light, between 8 a.m. and 4:00 p.m. The examiner stood behind the individual.

The instruments used for the oral examination were flat mouth mirrors N°5 and cotton tongs. Prior to the evaluation of the child, a rinse with water was performed and the clinical examination was carried out. The data were dictated to a trained annotator to fill out the epidemiological file.

After the clinical evaluation, visits were made to the homes of each child for the completion of the surveys through the mothers. The visits to the houses were made from 5 p.m., they visited from house to house, when the mother was not found, a time was set in which they were present (mother and son or daughter) and then returned. During the visit, the mother of the family responded to the surveys of determinants and social capital. After collecting the information from the surveys, the toothbrush holders were prepared with all the members of the families present, and cleaning kits were delivered for each member. To ensure the integrity of the selection and minimize response bias, dental hygiene kits were delivered as unannounced compensation upon completion of the entirety of data collection. In this way, the benefit did not act as an incentive for participation nor did it influence the process of select the sample.

### 2.6 Data analysis

Quantitative and qualitative descriptive analyses were performed to characterize the dataset. Inferential statistical analysis was conducted using Spearman’s rank correlation coefficient to assess the strength of relationships between variables, complemented by the coefficient of determination (R^2^) to evaluate predictive variance. All statistical procedures were executed using IBM SPSS Statistics software (version 27.0).

## 3 Results

This study was conducted in 12-year-old children (*n* = 61) and their respective families in the district of Pampacolca, Arequipa, Peru. The experience of dental caries was evaluated in the children, and the following variables were evaluated in the mothers: social determinants of health (SDOH) and social capital (SC).

The average number of decayed teeth in 12-year-olds was 5.23 ± 2.94, only 1 missing tooth was found, which on average is equivalent to 0.02 ± 0.12 missing teeth, and no filled teeth were found. The DMFT score for the 12-year-old child population of the district of Pampacolca, Arequipa was 5.25 ± 2.94 (Table 1).

**Table 1:** Dental caries experience in 12-year-old schoolchildren in the Pampacolca District - Arequipa.

	Dental caries experience							
	DT		MT		FT		DMFT	
Total	Mean	SD	Mean	SD	Mean	SD	Mean	SD
Total	5.23	2.94	0.02	0.12	0	0	5.25	2.94

DT, decay teeth; MT, missing teeth; FT, filled teeth; DMFT, sum of DT, MT, and FT.

Within the SDOH there are 7 indicators: gender, economic income, level of education, employment, housing conditions, environmental sanitation and access to health services. With respect to gender, of the total of 61 children, 37.7% (*n* = 23) were boys and 62.3% (*n* = 38) were girls. In terms of economic income, it was observed that 80.3% of the mothers (*n* = 49) received an economic income from some job, 83.6% (*n* = 51) of the mothers knew how to read and write, 78.7% of mothers (*n* = 48) did work, 91.8% (*n* = 56) of the families live in an independent house, 96.7% (*n* = 59) live in houses built with adobe on the walls, 67.2% (*n* = 41) of the families have their own houses fully paid, 93.4% (*n* = 57) have water pipes inside the house, 95.1% (*n* = 58) have water service every day and 39.3% (*n* = 24) have a public drainage network inside the house, 100% of families have electric lighting in their homes, 80.3% (*n* = 49) have 2 or 3 rooms in the house, where 27.9% (*n* = 17) of the families have 6 people living inside the house. Of the total of 61 children, 50.8% (*n* = 31) do not have any health insurance and 45.9% (*n* = 28) are affiliated with the SIS; for more details about SDOH in the study population see Supplementary Appendix 1.

The association between SDOH (as a whole) and dental caries experience was not statistically significant, explaining only 3.99% of the overall variance (*p* = 0.123). When analyzing individual determinants for the total population, only Employment showed a statistically significant association (*r* = 0.263, *p* = 0.040), although it explained only 6.91% of the variance. Other factors such as gender, income, and housing conditions did not show significant correlations (Table 2). The stratified analysis revealed more specific associations. In the group of schoolchildren with DMFT≤3, the Maternal Education Level showed a strong and statistically significant inverse correlation (*r* = −0.632, *p* = 0.007), explaining 39.94% of the variance in caries experience. This suggests that in children with lower disease severity, higher maternal education is a critical protective factor. Conversely, in the group with DMFT≥5, Access to Health Services emerged as the most relevant factor, showing a significant strong inverse correlation (*r* = −0.605, *p* = 0.001) and explaining 36.6% of the variance. Other determinants in these subgroups, including gender, income, and environmental sanitation, did not reach statistical significance (Table 2).

**Table 2:** Association between social determinants of health and dental caries experience in 12-year-old schoolchildren from the Pampacolca District – Arequipa.

	Dental caries experience								
SDOH (Social determinants of health)	DMFT			DMFT≤3			DMFT≥5		
	*r*	*r*^2^ (%)	*p*	*r*	*r*^2^ (%)	*p*	*r*	*r*^2^ (%)	*p*
Social determinants of health	0.200	3.99	0.123	−0.325	10.53	0.204	−0.068	0.46	0.736
Gender	0.229	5.25	0.760	0.469	21.97	0.058	0.010	0.01	0.961
Income	0.269	7.23	0.360	−0-051	0.26	0.845	−0.117	1.38	0.559
Education level	−0.048	0.23	0.712	−0.632	39.94	0.007*	0.017	0.03	0.931
Employment	0.263	6.91	0.040*	0.214	4.56	0.410	0.174	3.02	0.385
Housing conditions	0.025	0.06	0.845	−0.476	22.67	0.053	0.005	0.003	0.980
Environmental sanitation	0.050	0.25	0.699	−0.095	0.90	0.717	−0.292	8.50	0.140
Access to health services	−0.087	0.74	0.507	−0.320	10.26	0.210	−0.605	36.6	0.001*

*r*: Spearman’s Rho correlation; *r*^2^: coefficient of determination (%); *p*: statistical significance *p < 0.05.

The social capital variable is understood through 3 factors: Factor 1 (Cognitive Social Capital), Factor 2 (Structural Social Capital focused on citizenship) and Factor 3 (Structural Social Capital focused on membership). Regarding Factor 1, 83.6% (*n* = 51) consider that most people who live in their city cannot be trusted, 72.1% (*n* = 44) consider that most people in their city get along well with each other, 96.7% (*n* = 59) feel that they belong to their city and 62.3% (*n* = 38) feel that most people in their city would try to take advantage of them if they had the opportunity.

Regarding Factor 2, 63.9% (*n* = 39) have not joined other residents of their city to solve a problem or work together, 77% (*n* = 47) have not talked to local authorities or government representatives about anything or problem in Pampacolca; on Factor 3 of social capital, which includes having participated in some organization of the population and, on the other hand, having received some support from a human group; In this sense, only 8.2% (*n* = 5) actively participated in community organizations, 31.1% (*n* = 19) in food groups, 36% (*n* = 22) in political groups, and 23% (*n* = 14) in religious groups, 42.6% (*n* = 26) in sports groups, 6.6% (*n* = 4) in health groups, 39.3% (*n* = 24) in school committees, and 3.3% (*n* = 2) in vigilance committees. In addition, 39.3% (*n* = 24) received support from family members, only 19.7% (*n* = 12) received support from neighbors, 34.4% (*n* = 21) received support from friends who are not neighbors, only 8.2% (*n* = 5) received support from community leaders, 24.6% (*n* = 15) received support from religious leaders, 23% (*n* = 14) received support from political leaders, 65.6% (*n* = 40) received support from a government representative, 44.3% (*n* = 27) received support from a representative of the municipality, and 6.6% (*n* = 4) received support from a charitable organization; for more details of the social capital see Supplementary Appendix 2.

Regarding social capital as a whole, the coefficient of determination showed that it explained only 0.58% of the variance in dental caries experience for the total sample (*p* = 0.558). In the stratified analysis, social capital explained 8.24% of the variance in the DMFT≤3 group and 10.07% in the DMFT≥5 group; however, these relationships were not statistically significant (Table 3). When analyzing individual social capital factors, most associations showed low levels of explained variance and lacked statistical significance. For the total population, Factor 1 (Cognition), Factor 2 (Citizenship), and Factor 3 (Membership) explained 0.72, 2.76, and 0.11% of the variance, respectively. In the group of schoolchildren with DMFT≤3, Factor 1 (Cognition) showed a statistically significant moderate correlation (*r* = 0.494, *p* = 0.044), explaining 24.4% of the variance in caries experience. In contrast, Factor 2 and Factor 3 in this same group explained 4.77 and 16.27% of the variance, respectively, without reaching statistical significance. Finally, in the DMFT≥5 group, no social capital factors showed a significant association, with explained variances ranging from 1.93 to 3.98% (Table 3).

**Table 3:** Association between the social capital of families and the experience of dental caries in 12-year-old schoolchildren from the District of Pampacolca - Arequipa, Peru.

	Dental caries experience								
	DMFT			DMFT≤3			DMFT≥5		
Social capital	*r*	*r*^2^ (%)	*p*	*r*	*r*^2^ (%)	*p*	*r*	*r*^2^ (%)	*p*
SASCAT	−0.077	0.586	0.558	−0.287	8.24	0.264	0.317	10.07	0.107
Cognition	−0.085	0.72	0.513	0.494	24.40	0.044*	0.139	1.93	0.489
Citizenship	−0.166	2.76	0.200	−0.219	4.77	0.399	0.192	3.68	0.338
Membership	0.033	0.11	0.799	−0.403	16.27	0.108	0.200	3.98	0.318

*r*: Spearman’s Rho correlation; *r*^2^: coefficient of determination (%); *p*: statistical significance *p < 0.05.

## 4 Discussion

It was observed that the DMFT index was 5.23 (±2.54) in this study, a community index considered high for dental caries experience. Studies carried out in the city of Cusco, Peru (8, 9) in remote rural communities found DMFT of 6 and 8.6 respectively, coinciding with our results observed in a rural community in Arequipa, which could show that in Peruvian rural communities the disease develops in a similar way, taking into account the type of similar diet, socioeconomic level (10, 11) and above all, the lack of access to health services in remote rural communities (10, 11), which in order to access dental care must travel for many hours from their homes compared to urban areas where the access to dental, care and prevention programs should, in theory, decrease the prevalence of caries (9, 11).

Another study conducted in Brazil (12), where DMFT was evaluated in Brazilian rural communities, found that for the age of 12 years the index was 1.38 ± 2.02, results that differ from our results; however, it was observed that an index with more similar values was found in this same population, but from the age of 16 years (12), something very interesting if we compare the preventive measures at the community level used by Brazil and Peru, Brazil being a country with drinking water with fluoride, which slows down the demineralization process in a population that constantly receives water with fluoride promoting remineralization and that does not depend on the will of the population for its action, unlike Peru where there is no drinking water with fluorides, only salt, but it will depend on the preference for the preparation of meals in families and this strategy has not been shown to be effective in the country.

On the other hand, plaque disorganization is a habit that is practiced less in Peruvian children in rural areas compared to those who live in urban areas (4). This factor is important since dental caries disease is closely related to the control of biofilm.

According to our results related to the SDOH, it can be observed that, although the majority (80.3%) of the mothers who responded receive money from some type of job and 83.6% know how to read and write, 50% of their children do not have affiliation to any health insurance, a factor that has been shown to be related to the creation and perpetuation of inequities in oral health in children (13, 14).

Social capital has been highlighted as one of the main determinants of health according to the WHO (15), and in this study it can be observed that between 60 and 84% of the inhabitants of the district of Pampacolca feel that their neighbors are not trustworthy, since if they had the opportunity they would take advantage of them and would not work as a team which demonstrates a high degree of distrust and low social capital, especially of Factor 1 (cognitive social capital), which refers to social capital linked to an individual or group through relationships of mutual recognition, emphasizing the individual’s ability to use these resources to his or her advantage (16).

When we look at the results of social capital on the relationship with authorities, despite the fact that 96% have a high sense of belonging to their community and between 44 and 65% receive support from the government or the municipality, at the same time 77% would not turn to their authorities to solve a problem and only 8% participate in organizations in their community (6% in health organizations and 39.3% in school organizations). demonstrating that the concept of social capital as the concept of networks that facilitate coordinated actions to improve the community (7, 17), is not something that is practiced in this community. The high rate of experience of dental caries in the district of Pampacolca, province of Castilla, department of Arequipa; due to the results observed in the SDOH, but especially those found in social capital, this relationship is consistent with evidence suggesting a potential link where higher social capital tends to coincide with better oral health indicators (7, 18–20).

If we look at the study population in general, a statistically significant but weak correlation was found between employment and DMFT (*r* = 0.263, *p* = 0.040). In the DMFT≤3 group, the mother’s educational level explained 39.9% of the variance (*r* = −0.632, *p* = 0.007), while Factor 1 of social capital explained 24.4% of the variance (*r* = 0.494, *p* = 0.044). In the DMFT≥5 group, limited access to health services explained 36.6% of the variance (*r* = −0.605, *p* = 0.001), while no significant relationship was found with any social capital factor. These results may indicate that when groups are analyzed in a general way, the association with specific factors is masked; however, in different severities of dental caries experience, the associated factors appear to differ, which could be considered for public policy decisions to improve the oral health of this population. In the group with less experience of dental caries, the educational factor (21) is important, and in the group with high experience of dental caries, the factor of access to health services is the most preponderant (22).

This study has certain limitations, the population studied does not reflect the reality of Peru since on the contrary a rural population was chosen that has the characteristics of not having a dentist in the only health post, being more than 10 h from the city center, the main activity being agriculture and livestock farming. In addition, self-reporting instruments were used, which may be subject to bias. A potential limitation of this study is the absence of multivariate models for the adjustment of confounding variables. However, it is important to note that the sample was selected under criteria of high homogeneity: families with 12-year-old children, residents of an isolated rural community with equivalent socioeconomic levels and access to public services. This uniformity of the population reduces the likelihood that external factors such as age, geographic environment, or social status act as confounding variables, allowing the Spearman correlation to more directly reflect the association between the main variables in this specific context. On the other hand, while classic biological and behavioral factors, such as brushing frequency, free sugar intake, and fluoride exposure, are direct determinants of dental caries, this study deliberately focused on social determinants and social capital. It is recognized that the lack of measurement of these intermediate variables could limit the comprehensive view of the pathology; however, our interest was to explore how the social determinants and living conditions in an isolated rural community influence oral health, factors that are often omitted in traditional clinical studies.

Furthermore, given the cross-sectional nature of this study and the use of bivariate correlations, no temporal or causal relationships can be established. The associations found should be interpreted as co-occurrences within a specific context rather than direct causal links, as factors not measured in this study—such as specific dietary patterns—could also play a role.

In conclusion, the results suggest that specific social determinants and certain dimensions of social capital are associated with dental caries experience, with these associations varying according to the severity of the disease. In this group of 12-year-old children from a rural area, a greater experience of dental caries was linked to higher parental unemployment and limited access to health services. Conversely, a higher maternal educational level and cognitive social capital were associated with a lower experience of dental caries. These findings highlight the need for public health strategies that consider both structural factors and community networks, although the cross-sectional nature of this study precludes establishing causal relationships.

## Data Availability

The original contributions presented in the study are included in the article/Supplementary material, further inquiries can be directed to the corresponding authors.
