# Supplementary material for: Social inequalities in rural oral health: social capital and SDOH according to the severity of dental caries in Peru
Source: Front Public Health. 2026 May 29;14:1824635. doi: 10.3389/fpubh.2026.1824635 (PMC13259745; doi:10.3389/fpubh.2026.1824635)
Supplement: Supplementary file 1 [file Supplementary_File_1.docx]

**Appendix 1**

**Determinantes sociales de la salud en escolares de 12 años de edad del distrito de Pampacolca - Arequipa, Perú**

| Determinantes sociales de la salud | |  | | | N | | | | |  | | | % | | | | | | |  |
| --- | --- | --- | --- | --- | --- | --- | --- | --- | --- | --- | --- | --- | --- | --- | --- | --- | --- | --- | --- | --- |
| Género | |  | | |  | | | | |  | | | | | | | | | |  |
| Masculino | |  | | | 23 | | | | |  | | | 37.7 | | | | | | |  |
| Femenino | | | | | 38 | | | | |  | | | 62.3 | | | | | | |  |
|  | | | | |  | | | | |  | | | | | | | | | |  |
| Ingreso económico | | | | |  | | | | |  | | | | | | | | | |  |
| ¿Buscando trabajo habiendo trabajado antes? | | | | | 3 | | | | | 4.9 | | | | | | | | | |  |
| ¿Buscando trabajo por primera vez? | | | | | 0 | | | | | 0 | | | | | | | | | |  |
| ¿Estudiando y no trabajó? | | | | | 0 | | | | | 0 | | | | | | | | | |  |
| ¿Viviendo de su pensión y jubilaciòn y no trabajó? | | | | | 0 | | | | | 0 | | | | | | | | | |  |
| ¿Viviendo de sus rentas y no trabajó? | | | | | 2 | | | | | 3.3 | | | | | | | | | |  |
| ¿Al cuidado de su hogar y no trabajó? | | | | | 7 | | | | | 11.5 | | | | | | | | | |  |
| Otra | | | | | 49 | | | | | 80.3 | | | | | | | | | |  |
|  | | | | |  | | | | |  | | | | | | | | | |  |
| Nivel de educación | | | | |  | | | | |  | | | | | | | | | |  |
| Sabe leer y escribir | | | | |  | | | | |  | | | | | | | | | |  |
| Si | | | | | 51 | | | | | 83.6 | | | | | | | | | |  |
| No | | | | | 10 | | | | | 16.4 | | | | | | | | | |  |
|  | | | | |  | | | | |  | | | | | | | | | |  |
| Nivel de estudios | | | | |  | | | | |  | | | | | | | | | |  |
| Sin nivel | | | | | 2 | | | | | 3.3 | | | | | | | | | |  |
| Educación inicial | | | | | 0 | | | | | 0 | | | | | | | | | |  |
| Primaria | | | | | 26 | | | | | 42.6 | | | | | | | | | |  |
| Secundaria | | | | | 23 | | | | | 37.7 | | | | | | | | | |  |
| Superior no universitaria incompleta | | | | | 0 | | | | | 0 | | | | | | | | | |  |
| Superior no universitaria completa | | | | | 4 | | | | | 6.6 | | | | | | | | | |  |
| Superior universitaria incompleta | | | | | 4 | | | | | 6.6 | | | | | | | | | |  |
| Superior universitaria completa | | | | | 2 | | | | | 3.3 | | | | | | | | | |  |
| Empleo |  | | | | | | |  | | | |  | | | | | |  |  |  |
| Situación laboral |  | | | | | | |  | | | |  | |  | | | |  |  |  |
| No trabajó pero tenía trabajo | | | | | | | | 2 | | | |  | | 3.3 | | | |  |  |  |
| Aunque no trabajó, tiene algún negocio propio | | | | | | | | 0 | | | | 0 | | | | | |  |  |  |
| Realizó algún trabajo temporal por un pago de dinero o especie | | | | | | | | 1 | | | | 1.6 | | | | | |  |  |  |
| Estuvo ayudando en la chacra, tienda o negocio de un familiar sin pago alguno | | | | | | | | 1 | | | | 1.6 | | | | | |  |  |  |
| No trabajó | | | | | | | | 9 | | | | 14.8 | | | | | |  |  |  |
| Si trabajó | | | | | | | | 48 | | | | 78.7 | | | | | |  |  |  |
|  | | | | | | | |  | | | |  | | | | | |  |  |  |
| Condición laboral | | | | | | | |  | | | |  | | | | | |  |  |  |
| Empleado | | | | | | | | 17 | | | | 27.9 | | | | | |  |  |  |
| Obrero | | | | | | | | 11 | | | | 18 | | | | | |  |  |  |
| Trabajador independiente o por cuenta propia | | | | | | | | 24 | | | | 39.3 | | | | | |  |  |  |
| Empleador o patrono | | | | | | | | 0 | | | | 0 | | | | | |  |  |  |
| Trabajador familiar no remunerado | | | | | | | | 0 | | | | 0 | | | | | |  |  |  |
| Trabajador (a) del hogar | | | | | | | | 9 | | | | 14.8 | | | | | |  |  |  |
|  | | | | | | | |  | | | |  | | | | | |  |  |  |
| Condición de vivienda | | | | | | | |  | | | |  | | | | | |  |  |  |
| Tipo de vivienda | | | | | | | |  | | | |  | | | | | |  |  |  |
| Vivienda particular | | | | | | | |  | | | |  | | | | | |  |  |  |
| Casa independiente | | | | | | | | 56 | | | | 91.8 | | | | | |  |  |  |
| Departamento en edificio | | | | | | | | 0 | | | | 0 | | | | | |  |  |  |
| Vivienda en quinta | | | | | | | | 0 | | | | 0 | | | | | |  |  |  |
| Vivienda en casa de vecindad | | | | | | | | 4 | | | | 6.6 | | | | | |  |  |  |
| Choza o cabaña | | | | | | | | 0 | | | | 0 | | | | | |  |  |  |
| Vivienda improvisada | | | | | | | | 1 | | | | 1.6 | | | | | |  |  |  |
| Local no destinado para habitación humana | | | | | | | | 0 | | | | 0 | | | | | |  |  |  |
| Otro | | | | | | | | 0 | | | | 0 | | | | | |  |  |  |
| Vivienda colectiva | | | | | | | | 0 | | | | 0 | | | | | |  |  |  |
| Condiciones de ocupación de vivienda | | |  | | | | | |  | | | | | |  | | | | | |
| Ocupada | | |  | | | | | |  | | | | | |  | |  | | | |
| Con personas presentes | | | | | | | | | 61 | | | | | |  | | 100 | | | |
| Con personas ausentes | | | | | | | | | 0 | | | | | | 0 | | | | | |
| De uso ocasional | | | | | | | | | 0 | | | | | | 0 | | | | | |
| Desocupada | | | | | | | | | 0 | | | | | | 0 | | | | | |
|  | | | | | | | | |  | | | | | |  | | | | | |
| Material predominante de paredes de las viviendas | | | | | | | | |  | | | | | |  | | | | | |
| Ladrillo o bloque de cemento | | | | | | | | | 2 | | | | | | 3.3 | | | | | |
| Adobe o tapia | | | | | | | | | 59 | | | | | | 96.7 | | | | | |
| Madera, Quincha, estera, piedra con barro, piedra, sillar con cal o cemento u otro material | | | | | | | | | 0 | | | | | | 0 | | | | | |
|  | | | | | | | | |  | | | | | |  | | | | | |
| Material predominante de pisos de las viviendas  Tierra | | | | | | | | | 30 | | | | | | 49.2 | | | | | |
| Cemento | | | | | | | | | 25 | | | | | | 41 | | | | | |
| Losetas, terrazos, cerámicos o similares | | | | | | | | | 4 | | | | | | 6.6 | | | | | |
| Parquet o madera pulida, madera, laminas asfálticas, vinílicos o similares | | | | | | | | | 0 | | | | | | 0 | | | | | |
| Otro material | | | | | | | | | 2 | | | | | | 3.3 | | | | | |
|  | | | | | | | | |  | | | | | |  | | | | | |
| Tenencia de la vivienda | | | | | | | | |  | | | | | |  | | | | | |
| Alquilada | | | | | | | | | 11 | | | | | | 18 | | | | | |
| Propia por invasión | | | | | | | | | 0 | | | | | | 0 | | | | | |
| Propia pagándola a plazos | | | | | | | | | 4 | | | | | | 6.6 | | | | | |
| Propia totalmente pagada | | | | | | | | | 41 | | | | | | 67.2 | | | | | |
| Cedida por el centro de trabajo, otro hogar o institución | | | | | | | | | 5 | | | | | | 8.2 | | | | | |
| Otra forma | | | | | | | | | 0 | | | | | | 0 | | | | | |
| Saneamiento ambiental | | | |  | | |  | | | |  | | | | |  | | |  |  |
| Abastecimiento de agua | | | |  | | |  | | | |  | | | | |  | | |  |  |
| Red pública dentro de la vivienda (agua potable) | | | |  | | | 57 | | | |  | | | | | 93.4 | | |  |  |
| Red pública fuera de la vivienda pero dentro de la edificación (agua potable) | | | |  | | | 4 | | | |  | | | | | 6.6 | | |  |  |
| Pilón de uso público | | | |  | | | 0 | | | |  | | | | | 0 | | |  |  |
| Camión, cisterna u otro similar | | | |  | | | 0 | | | |  | | | | | 0 | | |  |  |
| Pozo | | | |  | | |  | | | |  | | | | | 0 | | |  |  |
| Río, acequia, manantial o similar | | | |  | | | 0 | | | |  | | | | | 0 | | |  |  |
| Vecino | | | |  | | |  | | | |  | | | | | 0 | | |  |  |
| Otro | | | |  | | | 0 | | | |  | | | | | 0 | | |  |  |
| Tenencia de servicios de agua de las viviendas | | | | | |  |  | | | |  | | | | |  | | |  |  |
| Si | | | | | |  | 58 | | | |  | | | | | 95.1 | | |  |  |
| No | | | | | |  | 3 | | | |  | | | | | 4.9 | | |  |  |
|  | | | | | |  |  | | | |  | | | | |  | | |  |  |
| Eliminación de excretas de las viviendas | | | | | |  |  | | | |  | | | | |  | | |  |  |
| Red pública de desagüe dentro de la vivienda | | | | | |  | 24 | | | |  | | | | | 39.3 | | |  |  |
| Red pública de desagüe fuera de la vivienda, pero dentro de la edificación | | | | | |  | 3 | | | |  | | | | | 4.9 | | |  |  |
| Pozo séptico | | | | | |  | 9 | | | |  | | | | | 14.8 | | |  |  |
| Pozo ciego o negro/ letrinha | | | | | |  | 0 | | | |  | | | | | 0 | | |  |  |
| Río, acequia o canal | | | | | |  | 2 | | | |  | | | | | 3.3 | | |  |  |
| No tiene | | | | | |  | 23 | | | |  | | | | | 37.7 | | |  |  |
|  | | | | | |  |  | | | |  | | | | |  | | |  |  |
| Tenencia de alumbrado eléctrico | | | | | |  |  | | | |  | | | | |  | | |  |  |
| Si | | | | | |  | 61 | | | |  | | | | | 100 | | |  |  |
| No | | | | | |  | 0 | | | |  | | | | | 0 | | |  |  |
|  | | | | | |  |  | | | |  | | | | |  | | |  |  |
| Número de habitaciones en las viviendas | | | | | |  |  | | | |  | | | | |  | | |  |  |
| 1 | | | | | |  | 4 | | | |  | | | | | 6.6 | | |  |  |
| 2 | | | | | |  | 23 | | | |  | | | | | 37.7 | | |  |  |
| 3 | | | | | |  | 26 | | | |  | | | | | 42.6 | | |  |  |
| 4 | | | | | |  | 4 | | | |  | | | | | 6.6 | | |  |  |
| 8 | | | | | |  | 4 | | | |  | | | | | 6.6 | | |  |  |
|  | | | | | |  |  | | | |  | | | | |  | | |  |  |
| Número de personas que habitan en las viviendas | | | | | |  |  | | | |  | | | | |  | | |  |  |
| 3 | | | | | |  | 15 | | | |  | | | | | 24.6 | | |  |  |
| 4 | | | | | |  | 5 | | | |  | | | | | 8.2 | | |  |  |
| 5 | | | | | |  | 11 | | | |  | | | | | 18 | | |  |  |
| 6 | | | |  | | | 17 | | | |  | | | | | 27.9 | | |  |  |
| 7 | | | |  | | | 1 | | | |  | | | | | 1.6 | | |  |  |
| 8 | | | |  | | | 10 | | | |  | | | | | 16.4 | | |  |  |
| 10 | | | |  | | | 2 | | | |  | | | | | 3.3 | | |  |  |
|  | | | |  | | |  | | | |  | | | | |  | | |  |  |
| Acceso a servicios de salud | | | |  | | |  | | | |  | | | | |  | | |  |  |
| SIS | | | |  | | | 28 | | | |  | | | | | 45.9 | | |  |  |
| EsSalud | | | |  | | | 2 | | | |  | | | | | 3.3 | | |  |  |
| Otro seguro | | | |  | | | 0 | | | |  | | | | | 0 | | |  |  |
| Ninguno | | | |  | | | 31 | | | |  | | | | | 50.8 | | |  |  |
