# Supplementary material for: Social inequalities in rural oral health: social capital and SDOH according to the severity of dental caries in Peru
Source: Front Public Health. 2026 May 29;14:1824635. doi: 10.3389/fpubh.2026.1824635 (PMC13259745; doi:10.3389/fpubh.2026.1824635)
Supplement: Supplementary file 2 [file Supplementary_File_2.docx]

**Appendix 2**

**Capital social cognitivo, estructural enfocado a ciudadanía, estructural enfocado a membresía en las familias de escolares de 12 años de edad del distrito de Pampacolca - Arequipa, Perú**

| **Tabla N°4.** | Capital social cognitivo de las familias de escolares de 12 años del distrito de Pampacolca - Arequipa | | | | | | | |
| --- | --- | --- | --- | --- | --- | --- | --- | --- |
|  |  |  |  |  |  |  |  |  |
|  |  |  |  |  |  |  |  |  |
|  |  |  |  |  |  |  |  |  |
|  |  |  |  |  |  |  |  |  |
| Factor 1 | ¿Se puede confiar en la mayoría de personas que viven en Pampacolca? | | ¿La mayoría de personas en Pampacolca se llevan bien entre ellos? | | ¿Se siente Ud. Que pertenece a Pampacolca? | | ¿Siente Ud. Que la mayoría de personas en Pampacolca tratarían de aprovecharse de Ud. Si ellos tuvieran la oportunidad? | |
|  |  |  |  |  |  |  |  |  |
|  |  |  |  |  |  |  |  |  |
|  |  |  |  |  |  |  |  |  |
|  |  |  |  |  |  |  |  |  |
|  | N | % | N | % | N | % | N | % |
| Si | 10 | 16.4 | 17 | 27.9 | 59 | 96.7 | 38 | 62.3 |
| No | 51 | 83.6 | 44 | 72.1 | 2 | 3.3 | 23 | 37.7 |
| Total | 61 | 100 | 61 | 100 | 61 | 100 | 61 | 100 |
|  |  |  |  |  |  |  |  |  |
| Factor 1: Capital Social cognitivo | | |  |  |  |  |  |  |

| **Tabla N°5.** | | | | **Capital social estructural enfocado a ciudadanía de las familias de escolares de 12 años de edad del distrito de Pampacolca - Arequipa** | | | | | | | | | | | | | | | | | | |  |  |  |
| --- | --- | --- | --- | --- | --- | --- | --- | --- | --- | --- | --- | --- | --- | --- | --- | --- | --- | --- | --- | --- | --- | --- | --- | --- | --- |
|  | | | |  |  |  |  |  |  |  |  |  |  |  |  |  |  |  |  |  |  |  |  |  |  |
| Factor 2 |  | | | ¿Se ha unido a otros residentes de Pampacolca para resolver un problema o hacer trabajos juntos? | | | | | | | | ¿Ha conversado con autoridades locales o representantes del gobierno acerca de alguna cosa o problema para Pampacolca? | | | | | | | | | | |  |  |  |
|  |  |  |  | N | | | % | | | | | N | | | | | % | | | | | |  |  |  |
| Si | | | | 22 | | | 36.1 | | | | | 14 | | | | | 23 | | | | | |  |  |  |
| No | | | | 39 | | | 63.9 | | | | | 47 | | | | | 77 | | | | | |  |  |  |
| Total | | | | 61 | | | 100 | | | | | 61 | | | | | 100 | | | | | |  |  |  |
|  | | | |  | | |  | | | | |  | | | | |  | | | | | |  |  |  |
| Factor 2: Capital Social estructural enfocado a ciudadanía | | | | | | | | | | | | | | | | | | | | | | |  |  |  |
|  | | | | | | | | | | | | | | | | | | | | | | |  |  |  |
| **Tabla N°6.** | | | **Capital social estructural enfocado a membresía (participación) de las familias de escolares de 12 años de edad del distrito de Pampacolca - Arequipa** | | | | | | | | | | | | | | | | | | | | | | |
|  | | |  |  |  |  |  |  |  |  |  |  |  |  |  |  |  |  |  |  |  |  |  |  |  |
|  | | |  |  | |  |  | |  |  |  |  | |  |  |  |  | |  |  |  |  |  | |  |
|  | | |  |  | |  |  | |  |  |  |  | |  |  |  |  | |  |  |  |  |  | |  |
|  | | |  |  | |  |  | |  |  |  |  | |  |  |  |  | |  |  |  |  |  | |  |
| Factor 3 (Participación) | | | Organizaciones comunitarias | | | Grupos alimenticios | | | Grupos políticos | | Grupos religiosos | | | Grupos deportivos | | Grupos de salud | | | Comités de colegio | | Comités de Vigilancia | | Otros | | |
|  |  |  |  |  |  |  |  |  |  |  |  |  |  |  |  |  |  |  |  |  |  |  |  |  |  |
|  |  |  | N | % | | N | % | | N | % | N | % | | N | % | N | % | | N | % | N | % | N | | % |
| Si | | | 5 | 8.2 | | 19 | 31.1 | | 22 | 36 | 14 | 23 | | 26 | 42.6 | 4 | 6.6 | | 24 | 39.3 | 2 | 3.3 | 4 | | 6.6 |
| No | | | 56 | 91.8 | | 42 | 68.9 | | 39 | 63.9 | 47 | 77 | | 35 | 57.4 | 57 | 93.4 | | 37 | 60.7 | 59 | 96.7 | 57 | | 93.4 |
| Total | | | 61 | 100 | | 61 | 100 | | 61 | 100 | 61 | 100 | | 61 | 100 | 61 | 100 | | 61 | 100 | 61 | 100 | 61 | | 100 |
|  | | |  |  | |  |  | |  |  |  |  | |  |  |  |  | |  |  |  |  |  | |  |
| Factor 3: Capital Social estructural enfocado a membresía | | | | | | | | | | | | | | | |  |  | |  |  |  |  |  | |  |

| **Tabla N°6a.** | **Capital social estructural enfocado a membresía (participación) de las familias de escolares de 12 años de edad del distrito de Pampacolca - Arequipa** | | | | | | | | | | | | | | | | | | | |
| --- | --- | --- | --- | --- | --- | --- | --- | --- | --- | --- | --- | --- | --- | --- | --- | --- | --- | --- | --- | --- |
|  |  |  |  |  |  |  |  |  |  |  |  |  |  |  |  |  |  |  |  |  |
|  |  |  |  |  |  |  |  |  |  |  |  |  |  |  |  |  |  |  |  |  |
|  |  |  |  |  |  |  |  |  |  |  |  |  |  |  |  |  |  |  |  |  |
| Factor 3 (Apoyo) | Familias | | Vecinos | | Amigos que no son vecinos | | Dirigentes de la comunidad | | Dirigentes religiosos | | Líderes políticos | | Representantes del gobierno | | Representantes de la municipalidad | | De una organización de caridad /ONG | | Otros | |
|  |  |  |  |  |  |  |  |  |  |  |  |  |  |  |  |  |  |  |  |  |
|  |  |  |  |  |  |  |  |  |  |  |  |  |  |  |  |  |  |  |  |  |
|  | N | % | N | % | N | % | N | % | N | % | N | % | N | % | N | % | N | % | N | % |
| Si | 24 | 39.3 | 12 | 19.7 | 21 | 34.4 | 5 | 8.2 | 15 | 24.6 | 14 | 23 | 40 | 65.6 | 27 | 44.3 | 4 | 6.6 | 4 | 6.6 |
| No | 37 | 60.7 | 49 | 80.3 | 40 | 65.6 | 56 | 91.8 | 46 | 75.4 | 47 | 77 | 21 | 34.4 | 34 | 55.7 | 57 | 93.4 | 57 | 93.4 |
| Total | 61 | 100 | 61 | 100 | 61 | 100 | 61 | 100 | 61 | 100 | 61 | 100 | 61 | 100 | 61 | 100 | 61 | 100 | 61 | 100 |
|  |  |  |  |  |  |  |  |  |  |  |  |  |  |  |  |  |  |  |  |  |
| Factor 3: Capital Social estructural enfocado a membresía | | | | | | | | | |  |  |  |  |  |  |  |  |  |  |  |
